# Modeling of a Neural System Based on Statistical Mechanics

**DOI:** 10.3390/e20110848

**Published:** 2018-11-05

**Authors:** Myoung Won Cho, MooYoung Choi

**Affiliations:** 1 Department of Global Medical Science, Sungshin Women’s University, Seoul 01133, Korea; 2 Department of Physics and Astronomy and Center for Theoretical Physics, Seoul National University, Seoul 08826, Korea

**Keywords:** neural network model, statistical mechanics, free-energy minimization principle

## Abstract

The minimization of a free energy is often regarded as the key principle in understanding how the brain works and how the brain structure forms. In particular, a statistical-mechanics-based neural network model is expected to allow one to interpret many aspects of the neural firing and learning processes in terms of general concepts and mechanisms in statistical physics. Nevertheless, the definition of the free energy in a neural system is usually an intricate problem without an evident solution. After the pioneering work by Hopfield, several statistical-mechanics-based models have suggested a variety of definition of the free energy or the entropy in a neural system. Among those, the Feynman machine, proposed recently, presents the free energy of a neural system defined via the Feynman path integral formulation with the explicit time variable. In this study, we first give a brief review of the previous relevant models, paying attention to the troublesome problems in them, and examine how the Feynman machine overcomes several vulnerable points in previous models and derives the outcome of the firing or the learning rule in a (biological) neural system as the extremum state in the free energy. Specifically, the model reveals that the biological learning mechanism, called spike-timing-dependent plasticity, relates to the free-energy minimization principle. Basically, computing and learning mechanisms in the Feynman machine base on the exact spike timings of neurons, such as those in a biological neural system. We discuss the consequence of the adoption of an explicit time variable in modeling a neural system and the application of the free-energy minimization principle to understanding the phenomena in the brain.

## 1. Introduction

A neural network is a specific system to task with computing, memorizing, and thinking. The history of neural network modeling unfolded with the work of McCulloch and Pitts [1]. They introduced the notion of a formal neuron as a two-state threshold element and showed how a network of such elements can perform logical calculations. Their work paved the development of the artificial neuron models which are designed to mimic aspects of biological counterparts. Meanwhile, the first biological neuron model, introduced by Hodgkin and Huxley [2], expresses how the action potential in a neuron is initiated and propagates according to a set of nonlinear differential equations that approximate the electrical characteristics of a neuron. While artificial neurons usually take continuous values of activity on the discrete time domain, biological neurons give off discrete firing pulses on the continuous time domain. A firing-rate neuron model, which can be described as a low-pass filtered version of the biological neuron model, makes the bridge between the activity rule in the artificial neuron model and the firing dynamics in the biological neuron model. However, the firing-rate model, based on the neural activities on the time scale of tens of milliseconds or the moving average of firing rate, may not account for aspects of spike timings and correlations on the millisecond time scale.

The learning process is another important dynamics of a neural system. A neural system can memorize or learn via changing synaptic strengths, depending on neural activities. According to the basic mechanism for synaptic plasticity proposed by Hebb [3], an increase in synapse efficacy arises from the presynaptic cell’s repeated and persistent stimulation of the postsynaptic cell. The resulting Hebbian theory attempts to explain associative learning, in which simultaneous activation of cells leads to a pronounced increase in the synaptic strength between those cells and provides a biological basis for errorless learning methods for education and memory rehabilitation. A firing-rate model, which is an artificial neuron model, describes the increase in the synaptic connection strength as a function of pre- and post-synaptic neural activities, e.g., as the product of the activities. On the other hand, series of experiments have revealed that the plasticity of a biological synapse occurs depending on the spike correlations on the millisecond time scale [4,5]. The learning behavior in a biological neural network could take after a learning rule in an artificial neuron model if spikes have weak correlations in the high-frequency regime; however, the biological learning mechanism, called the spike-timing-dependent plasticity (STDP) rule, can provide the system with more plentiful and useful learning behavior. One of them is the execution of competitive learning behavior, which is necessary for the development of a neural network with adaptive functions. Traditionally, inhibitory connections between neurons are believed to be essential for the execution of competitive learning behavior in a neural network. In contrast, the STDP rule can introduce competitive learning behavior to a neural network without inhibitory connections [6,7,8,9].

Meanwhile, neurons are inherently probabilistic in responding to external inputs, owing to the presence of noisy currents or chaotic behaviors associated with nonlinearity. Some neuron models describe the stochastic firing dynamics through differential equations with stochastic terms or Langevin dynamic equations; some other models do this through the Markov-chain Monte Carlo processes. Further, they describe the firing rule and/or the learning rule via statistical mechanism, and facilitate the use of statistical mechanics, which aims to explain the measurable properties of macroscopic systems on the basis of the behavior of the microscopic constituents of those systems. While an Ising-type model, well known to describe phase transitions in statistical mechanics, was first proposed by Little [10,11], a landmark was presented by Hopfield, who suggested a fruitful analogy between the asymptotic dynamics of such networks and equilibrium properties of random magnetic systems [12]. His work paved the road to the introduction of concepts and methods of statistical mechanics of random physical systems to the study of neural networks. Further, there are suggestions that the minimization of a free-energy may be the key principle in understanding how the brain works and how the brain structures form [13].

A statistical-mechanics-based model usually describes the firing rule and/or the learning rule as Markov-chain Monte Carlo processes of an energy function or gradient descents in a free energy. The free energy or the entropy of a neural model system is often defined intuitively as an objective quantity in information theory [13] (cf. Section 3.3). From the viewpoint of statistical physics, however, how to define a proper (free) energy in a complex and open system is far from a plain problem with an evident solution. One of the stringent problems is that an energy function should satisfy the invariance condition under the exchange of element positions, so that interaction strength between two elements should be symmetric. Some of the existing models solve the problem by assuming that all connections are symmetric (Section 3.1 and Section 3.2), by treating asymmetric connections as a factor in external inputs (Section 3.4), or by estimating the entropy from the firing covariance in a Langevin dynamic equation (Section 3.4). If the energy and possible configurations of a system are well defined, the free energy of the system may be derived strictly from the internal energy and the entropy. Then, the minimization of the free energy brings about the minimization of the internal energy and/or the maximization of the entropy, where the dominance of either part is controlled by the temperature. The internal energy reaches its minimum at the extremum state, which is usually determined by recursive interactions between neurons or synaptic connections. The entropy relates to random fluctuations of firing states from the extremum state. Such random fluctuations in the firing dynamics and/or the learning dynamics is often adopted to avoid trapping in local minima during the learning process. On the other hand, some models make use of the entropy maximization principle as the key mechanism to invest a neuron model system with adequate competitive relationship between synaptic connections (Section 3.3 and Section 3.4).

In the Feynman machine, the free energy is defined via the path integral, rather than from the Boltzmann distribution. Adopting asymmetric connections as well as asymmetric interactions between neurons in the time direction, the path integral formulation fulfils the invariance condition. Then, minimization of the free energy introduces a proper competitive learning rule to the Feynman machine. In particular, the biological synaptic plasticity rule is derived from the free-energy minimization principle. Attributes of firing dynamics or learning dynamics in a Feynman machine can resemble those in a conventional neuron model in a limit condition. However, the Feynman machine utilizes the exact timing of neural firings as the essential quantity in the implementation of computing or learning mechanism, such as biological neurons performing a function or communication through the use of the sensitivity for spike timings on the millisecond time scale [14,15].

## 2. Prologue

A firing-rate model expresses neural dynamics in terms of continuous variable $u_{i}\left(t\right)$, which represents the moving average of neural firings, i.e., the firing rate of neuron *i*. The time-dependent firing rate is often modeled as

(1) $$\begin{array}{c} \tau \frac{du_{i}}{dt}=-u_{i}+f\left(v_{i}\right), \end{array}$$

where τ is the relaxation time and *f* is the activation function, with $v_{i}$ being the scaled membrane potential of neuron *i*. If neuron *i* receives synaptic currents from neuron *j* via connection $W_{ij}$ and receives external input $h_{i}$, the scaled membrane potential is given by the sum of synaptic currents and external inputs:

(2) $$\begin{array}{c} v_{i}=\sum_{j}W_{ij}u_{i}+h_{i}. \end{array}$$

At the steady state, defined by $du_{i}/dt=0$, the firing rate takes the simple form $u_{i}=f\left(v_{i}\right)$, which corresponds to the activity rule in an artificial neuron model. According to the simple Hebbian rule, the change of the connection strength is given by the product of the activities of pre- and post-synaptic neurons:

(3) $$\begin{array}{c} \Delta W_{ij}\propto u_{i}u_{j}. \end{array}$$

**Figure 1:**
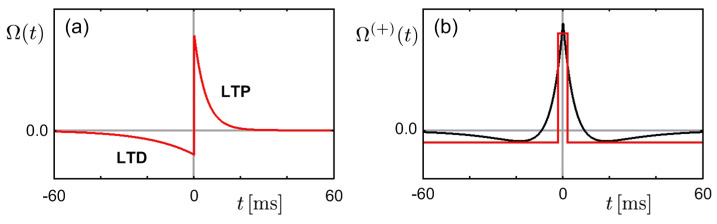
(**a**) Plot of a STDP window Ω(t), given by $\Omega \left(t\right)=Ae^{-t/2}$ for t>0 and $-\left(A/4\right)e^{t/20}$ for t<0 with a constant *A*. (**b**) Plot of the even part $\Omega^{\left(+\right)}$, given by $\Omega^{\left(+\right)}=A$ for |t|≤2 and −A/8 otherwise (red line) and by $\Omega^{\left(+\right)}=\frac{1}{2}\left[\Omega \left(t\right)+\Omega \left(-t\right)\right]$ with $\Omega \left(t\right)=1.5Ae^{-t/2}$ for t>0 and $-\left(1.5A/4\right)e^{t/20}$ for t<0 (black line).

Meanwhile, a two-state model or a spike-timing-based model expresses neural dynamics in terms of binary variable $\phi_{i}\left(t\right)$, which can only take values of 0 and 1. In the case of discrete time representation, $\phi_{i}\left(t\right)$ specifies the firing state of neuron *i* at time *t*; in the continuous time representation, it measures the number of firings in a small time interval. A biological synapse changes its strength according to the spike-timing-dependent plasticity (STDP) rule:

(4) $$\begin{array}{c} \Delta W_{ij}=\Omega_{ij}\left(t-t^{\prime }\right)\phi_{i}\left(t\right)\phi_{j}\left(t^{\prime }\right), \end{array}$$

where the STDP window $\Omega_{ij}\left(t\right)$ determines the change of the connection strength $W_{ij}$ depending on the difference between the firing timings of post- and pre-synaptic neurons. On a long-time scale, the connection strength change is given by

(5) $$\begin{array}{c} \Delta W_{ij}=\int dt\;\Omega_{ij}\left(t\right)\;C_{ij}\left(t\right), \end{array}$$

where $C_{ij}\left(t-t^{\prime }\right)=\langle \phi_{i}\left(t\right)\phi_{j}\left(t^{\prime }\right)\rangle$ is the cross-correlation function of neurons *i* and *j*. In general, the STDP window produces long-term potentiation (LTP) for pre-before-post pairings and long-term depression (LTD) for post-before-pre pairings (see Figure 1a). It often has a second interval of depression for pre-before-post pairings. The LTP part leads to an increase in the synaptic strength for a causally correlated neural firing pair. On the other hand, the LTD part, bringing about a decrease in the synaptic strength for a neural firing pair with weak causality, may introduce the competitive learning behavior in the absence of inhibitory connections. The even part and the odd part of the STDP window, $\Omega_{ij}^{\left(\pm \right)}\equiv \frac{1}{2}\left[\Omega_{ij}\left(t\right)\pm \Omega_{ij}\left(-t\right)\right]$, determine the changes in the sum of and in the difference between reciprocal connections, $W_{ij}^{\left(\pm \right)}\equiv \frac{1}{2}\left(W_{ij}\pm W_{ji}\right)$, respectively, as follows:

(6) $$\begin{array}{c} \Delta W_{ij}^{\left(\pm \right)}=\int dt\;\Omega_{ij}\left(t\right)\;C_{ij}^{\left(\pm \right)}\left(t\right)=\int dt\;\Omega_{ij}^{\left(\pm \right)}\left(t\right)\;C_{ij}\left(t\right) \end{array}$$

with $C_{ij}^{\left(\pm \right)}\equiv \frac{1}{2}\left[C_{ij}\left(t\right)\pm C_{ji}\left(t\right)\right]=\frac{1}{2}\left[C_{ij}\left(t\right)\pm C_{ij}\left(-t\right)\right]$. Namely, the change of the symmetric connection strength $W_{ij}^{+}$ is determined by the even part of the STDP window. Heretofore, the symmetric connection strength is denoted by $J_{ij}$; a function of such symmetric connection strengths could serve as an energy function.

## 3. Statistical-Mechanics-Based Models

### 3.1. Hopfield Network

A Hopfield network consists of a group of neurons together with symmetric connections between them. The state of neuron *i* is represented by the variable $\sigma_{i}\equiv 2\phi_{i}-1$, which takes values ±1. The connection strengths between neurons are symmetric ($J_{ij}=J_{ji}$) and have no self-connection ($J_{ii}=0$). In the learning process, the Hopfield network memorizes a set of *p* binary patterns $\left\{\sigma^{\left(n\right)}\right\}$ (n=1,2,…,p) with $\sigma_{i}^{\left(n\right)}\in \left\{-1,1\right\}$, which is achieved by setting the connections as the sum of outer products:

(7) $$\begin{array}{c} J_{ij}=\left\{\begin{array}{cc} \eta \sum_{n=1}^{p}\sigma_{i}^{\left(n\right)}\sigma_{j}^{\left(n\right)} & \;\;\mathrm{for}\;i\neq j\\ 0 & \;\;\mathrm{for}\;i=j \end{array}\right. \end{array}$$

with the constant η specifying the overall connection strength. In the firing process, the state of each neuron is updated according to the following rule

(8) $$\begin{array}{c} \sigma_{i}\left(t+1\right)=\left\{\begin{array}{cc} +1 & \;\;\mathrm{if}\;\sum_{j}J_{ij}\sigma_{i}\left(t\right)+B_{i}\geq 0\\ -1 & \;\;\mathrm{otherwise} \end{array}\right., \end{array}$$

where the constant $B_{i}$ controls the bias of neuron *i*. Namely, the state of neuron *i* is updated as if the neuron had a threshold activation function with the threshold $-B_{i}$. The Hopfield network possesses the desired memory patterns $\left\{\sigma^{\left(n\right)}\right\}$ as its attractors, so that it usually flows to one of them depending on its initial state.

It is of interest that the update rule can be understood as the single spin-flip sampling in an Ising model system with the energy function

(9) $$\begin{array}{c} E=-\frac{1}{2}\sum_{i,j}J_{ij}\sigma_{i}\sigma_{j}-\sum_{i}B_{i}\sigma_{i}. \end{array}$$

Whereas the deterministic update rule in Equation (8) corresponds to the zero-temperature dynamics, one may generalize it easily to finite temperatures by introducing probabilities. Specifically, adopting the importance sampling Markov process or the Monte Carlo method, we consider the probabilistic update in such a way that the probability of flipping $\sigma_{i}$ is given by $P\left(\sigma_{i}\to -\sigma_{i}\right)\propto \exp\left(-\Delta E_{i}/T\right)$, where $\Delta E_{i}$ is the energy difference in flipping of $\sigma_{i}$. It is obvious that the important sampling process reduces to the deterministic update rule in Equation (8) as the temperature *T* approaches zero.

It is also of interest to note that only a single neuron is updated at each time step. Such asynchronous dynamics contrasts with the synchronous dynamics where all the neurons are updated simultaneously [11]. Neither dynamics provides a very realistic description of the dynamics in real networks, which presumably lies in between. As an attempt toward a more realistic description of the dynamics, a dynamic model working in continuous time but taking into account relevant time scales was also presented [16].

### 3.2. Boltzmann Machine

The Boltzmann machine, which is often referred to as a Monte Carlo version of the Hopfield network [17], has the energy function given by

(10) $$\begin{array}{c} E\left[\phi \right]=-\frac{1}{2}\sum_{i,j}J_{ij}\phi_{i}\phi_{j}-\sum_{i}B_{i}\phi_{i}, \end{array}$$

where $\phi_{i}$ can only take values of 0 and 1 and $B_{i}=h_{i}+\theta_{i}$ with $h_{i}$ being the external input to neuron *i* and $\theta_{i}$ the negative of the activation threshold in the system running freely without external inputs. The neurons are divided into “visible” and “hidden” ones. The external input $h_{i}$ is applicable only if neuron *i* is a visible one and the system is in the training phase. In the case of the Metropolis importance sampling [18], the state of each neuron flips with the probability

(11) $$\begin{array}{c} P\left(\phi_{i}\to 1-\phi_{i}\right)=\min\left[e^{-\Delta E_{i}/T},1\right], \end{array}$$

where $\Delta E_{i}$ is the energy difference in the flipping of $\phi_{i}$. The expectation value of $\phi_{i}$ in the Monte Carlo process then obtains the form

(12) $$\begin{array}{c} \langle \phi_{i}\rangle =\frac{1}{Z}\mathrm{Tr}\;\phi_{i}\;e^{-E[\phi ]/T}, \end{array}$$

where $Z=\mathrm{Tr}\;e^{-E[\phi ]/T}$ is the partition function with the trace standing for the summation over all configurations ($\mathrm{Tr}\equiv \prod_{k}\sum_{\phi_{k}=0,1}$). The expectation value can also be obtained from the derivative of the partition function with respect to the external source in the following way:

(13) $$\begin{array}{c} \langle \phi_{i}\rangle =\frac{T}{Z}\frac{\partial Z}{\partial B_{i}}=-\frac{\partial F}{\partial B_{i}}, \end{array}$$

where F=−TlogZ is the free energy of the system. Similarly, the cross-correlation function between two neurons *i* and *j* reads

(14) $$\begin{array}{c} \langle \phi_{i}\phi_{j}\rangle =\frac{1}{Z}\mathrm{Tr}\;\phi_{i}\phi_{j}\;e^{-E[\phi ]/T}=\frac{T^{2}}{Z}\frac{\partial^{2}Z}{\partial B_{i}\partial B_{j}}. \end{array}$$

The learning rule for the Boltzmann machine is expressed originally in the form of a gradient descent process

(15) $$\begin{array}{c} \Delta J_{ij}\propto -\frac{\partial L}{\partial J_{ij}}, \end{array}$$

where *L* is the relative entropy or Kullback–Leibler (KL) divergence given by

(16) $$\begin{array}{c} L=\mathrm{Tr}\;P\left(\phi \right)\log\frac{P(\phi )}{P_{0}\left(\phi \right)}. \end{array}$$

The KL divergence measures the distance between probability distributions $P_{0}\left(\phi \right)$ and P(ϕ), vanishing if and only if the two distributions are identical. Here, $P\left(\phi \right)\propto e^{-E[\phi ]/T}$ is the probability of the state $\phi =\{\phi_{k}\}$ of visible neurons when the system is in the training phase. $P_{0}{\left(\phi \right)\equiv P\left(\phi \right)|}_{h=0}$ is the corresponding probability for the network running freely with no external input. After some calculation, the change in a connection strength is obtained as

(17) $$\begin{array}{c} \Delta J_{ij}\propto C_{ij}-C_{0,ij}, \end{array}$$

where the correlation function $C_{ij}\equiv \langle \phi_{i}\phi_{j}\rangle$ describes the average probability of two neurons both being in the *on* state with the environment clamping the states of the visual units, and $C_{0,ij}\equiv C_{ij}{|}_{h=0}$ is the corresponding probability for the network running freely without external inputs.

Interestingly, there is similarity between the learning rules based on the gradient descent in the KL divergence and in the free energy. Applying the density-matrix formalism with the correlation function taken as the density matrix $C=\{C_{ij}\}$, we express the free energy of the Boltzmann machine as F=U−TS with the internal energy $U=-\frac{1}{2}\sum_{i,j}J_{ij}C_{ij}-\sum_{i}B_{i}\rho_{i}$ and the entropy S=−trClogC, where $\rho_{i}\equiv \langle \phi_{i}\rangle$ stands for the firing probability of neuron *i*. With the condition $\sum_{j}J_{ij}\rho_{j}+B_{i}=0$ in the extremum state, the gradient descent in the free energy takes the form

(18) $$\begin{array}{c} \Delta J_{ij}\propto C_{ij}+T\frac{\partial S}{\partial J_{ij}}-\frac{1}{2}\sum_{k,l}J_{kl}\left[\frac{\partial C_{kl}}{\partial J_{ij}}-2\rho_{k}\frac{\partial \rho_{l}}{\partial J_{ij}}\right]. \end{array}$$

Note that the derivative of the entropy with respect to the connection strength does not depend on external inputs.

Further, there is another learning rule for the Boltzmann machine, which is a simplified version of the STDP rule [9]. It has been developed to explore the emergent structure in a neural network running freely, motivated by the report that the STDP rule leads to the development of small-world and scale-free graphs in simulations [19,20]. As well known, small-world and scale-free properties are ubiquitous in complex networks [21,22]. Conventional models explain that a small-world network, characterized by short path lengths and high clustering, emerges as a result of randomly replacing a fraction of links on a lattice by new, randomly chosen links [23] and that a scale-free network emerges from stochastic growth in which new nodes are added continuously and attach themselves preferentially to existing nodes [24]. However, there are such empirical networks as the brain network, possessing small-world and/or scale-free properties, to which the conventional models are not applicable. Specifically, a neural network with a static number of neurons acquires the scale-free properties not as a result of the preferential attachment or rich-get-richer process in a growing network but as an equilibrium state of the connecting–disconnecting processes. In the biological learning rule, the connecting and the disconnecting processes are related to the LTP and the LTD parts of the STDP window, respectively.

**Figure 2:**
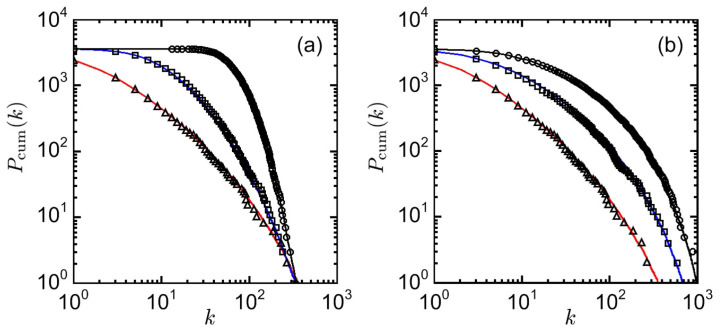
Log-log plots of the cumulative degree distribution $P_{\mathrm{cum}}\left(k\right)\equiv \sum_{k^{\prime }=k}^{\infty }P\left(k^{\prime }\right)$, with the degree distribution P(k) in a neural network, for different values of parameters: (**a**) competition strength η (=20, 10, and 5 for triangles, squares, and circles, respectively); and (**b**) wiring propensity μ (=17T, 19T, and 21T for triangles, squares, and circles, respectively). Lines are least-square fits of the data.

In the model, neurons flip their states according to the importance sampling rule in Equation (11). Simultaneously, the strength of each connection, which can take values *J* and 0, changes with the probability

(19) $$\begin{array}{c} P\left(J_{ij}\to J-J_{ij}\right)=\frac{1}{\tau }\min\;\left[e^{-\Delta F_{ij}/T},1\right], \end{array}$$

where τ controls the ratio of time scales in the firing and the learning process and $\Delta F_{ij}$ is the energy difference associated with flipping $J_{ij}$. The energy F in the learning process is given by

(20) $$\begin{array}{c} F=-\frac{1}{2}\sum_{i,j}\left(C_{ij}-\eta \rho_{i}\rho_{j}+\mu \right)J_{ij}, \end{array}$$

where the cross-correlation $C_{ij}$ and the firing probability $\rho_{i}\equiv \langle \phi_{i}\rangle$ are measured in a moving time window (of about 100 steps). The competition strength η controls the contributions of interactions relative to those of independent activations; μ is the wiring propensity (which is opposite to the wiring cost). The value of *J* is chosen to be sufficiently larger than *T* and the value of τ to be a small number as usual. Figure 2 shows the log-log plots of the cumulative degree distribution in neural networks, which exhibit the typical features of scale-free graphs.

In the above, the connection strength has been taken to be a binary number for convenience in Monte Carlo simulations and in the discriminant of connected and disconnected neural pairs. In the case that the connection strength takes continuous values, the learning process could be expressed as the gradient descent in the energy function:

(21) $$\begin{array}{c} \Delta J_{ij}\propto -\frac{\partial F}{\partial J_{ij}}=C_{ij}-\eta \rho_{i}\rho_{j}+\mu +\frac{1}{2}\sum_{k,l}J_{kl}\left[\frac{\partial C_{kl}}{\partial J_{ij}}-2\eta \rho_{k}\frac{\partial \rho_{l}}{\partial J_{ij}}\right], \end{array}$$

which is similar to the learning process in Equation (17) or Equation (18). The form of $\partial F/\partial J_{ij}$ originates from an approximate of the even part of the STDP window (see Figure 1b). Note also that F often serves as a free energy rather than energy. Henceforth, it is demonstrated that the biological synaptic plasticity rule can be interpreted as the minimization process for a free energy (see Section 5).

### 3.3. Informatix Rule

The informatix rule does not employ the definition of the energy or free energy of a neural system. Nevertheless, in view of informatics, it provides an important hint as to how the entropy maximization principle admits a stochastic neural network with competitive learning behavior. Consider a neural network composed of two input–output layers and only feedforward connections from input to output neurons. Suppose that the activity of neuron *i* is given by $u_{i}=f\left(v_{i}\right)$ with the scaled membrane potential $v_{i}$ given by Equation (2), where the feedforward connection $W_{ij}$ is relevant only for $i\in G_{o}$ and $j\in G_{i}$ with $G_{i}$ (or $G_{o}$) denoting the set of input (or output) neurons, and the external input $h_{i}$ is applicable only for $i\in G_{i}$. According to the informatics, the information transfer is measured by the joint entropy

(22) $$\begin{array}{c} H(u)=-\int DuP(u)\log P(u) \end{array}$$

with $\int Du\equiv \prod_{k}\int_{-\infty }^{\infty }du_{k}$. The joint entropy can be rewritten as

(23) $$\begin{array}{c} H\left(u\right)=\sum_{i\in G_{o}}H\left(u_{i}\right)-I\left(u\right), \end{array}$$

where $H(u_{i})$ is the marginal entropy of the output $u_{i}$ and I(u) the mutual information of the outputs. They obtain the form

(24) $$\begin{array}{c} H\left(u_{i}\right)=-P\left(u_{i}\right)\log P\left(u_{i}\right) \end{array}$$

and

(25) $$\begin{array}{c} I\left(u\right)=\int Du\;P\left(u\right)\log\frac{P(u)}{\prod_{i\in G_{o}}P\left(u_{i}\right)}. \end{array}$$

In informatics, an ideal learning process is to maximize the joint entropy or the information transfer from input to output neurons. Provided that the input and the output layers have the same number of neurons, the derivative of the joint entropy with respect to the connection strength is given by

(26) $$\begin{array}{c} \frac{\partial H(u)}{\partial W_{ij}}=\frac{\partial I(u)}{\partial W_{ij}}=\frac{\partial }{\partial W_{ij}}\mathrm{tr}\log\left(J\right), \end{array}$$

where other terms are assumed to be independent of *W* and $J_{ij}=\partial u_{i}/\partial u_{j}$ is the Jacobian of the information transfer from neuron *j* to neuron *i*. When there is no lateral connection between output neurons, the Jacobian would become $J_{ij}=W_{ij}\left(\partial u_{i}/\partial v_{i}\right)$. Finally, the learning rule maximizing the joint entropy reads

(27) $$\begin{array}{c} \Delta W_{ij}=\frac{\partial }{\partial W_{ij}}\mathrm{tr}\log\left(W\right)+\frac{\partial }{\partial W_{ij}}\sum_{k}\log\frac{\partial u_{k}}{\partial v_{k}}, \end{array}$$

where $\partial \mathrm{tr}\log\left(W\right)/\partial W_{ij}$ reduces to ${\left(W^{-1}\right)}_{ji}$ with the inverse matrix $W^{-1}$. The learning rule described by Equation (27) corresponds to the competition mechanism in the PSL model or in the Feynman machine, as shown in the following sections. A further manipulation of this equation gives the informatix rule [25], which is in turn related to the independent component analysis (ICA), a popular algorithm for blind source separation [26].

### 3.4. Pseudo-Stochastic Learning Model

Consider a neural network composed of input–output layers, where output neurons have feedforward connections *W* from input neurons and lateral connections *J* with other output neurons. Neglecting the activation function *f* or assuming $u_{i}=v_{i}$, we write the time-dependent firing rate in the form

(28) $$\begin{array}{c} \tau \frac{du_{i}}{dt}=-u_{i}+\sum_{j}J_{ij}u_{j}+\sum_{j}W_{ij}u_{j}+h_{i}, \end{array}$$

where the lateral connection $J_{ij}$ is applicable only for $i,j\in G_{o}$, the feedforward connection $W_{ij}$ only for $i\in G_{o}$ and $j\in G_{i}$, and the external input $h_{i}$ only for $i\in G_{i}$.

At stationarity ($du_{i}/dt=0$), Equation (28) gives the neural activity in the form

(29) $$\begin{array}{c} u_{i}=\left\{\begin{array}{cc} h_{i} & \;\mathrm{for}\;i\in G_{i}\\ \sum_{k,l}K_{ik}W_{kl}h_{l} & \;\mathrm{for}\;i\in G_{o}, \end{array}\right. \end{array}$$

where $K_{ij}$ is the recursive lateral interaction, given by a component of the matrix $K\equiv {\left(I-J\right)}^{-1}$ with *I* being the identity. Namely, letting D≡I−J, we have $K_{ij}=D_{ij}^{-1}={\left(I+J+J^{2}+\cdots \right)}_{ij}$. Adopting the simple Hebbian rule in Equation (3), the feedforward connection changes as

(30) $$\begin{array}{c} \Delta W_{ij}\propto \sum_{k,l}K_{ik}W_{kl}h_{l}h_{j}. \end{array}$$

In consideration of the learning process under varying external inputs, we write Equation (30) in the form

(31) $$\begin{array}{c} \Delta W_{ij}\propto {\left(KWQ\right)}_{ij}, \end{array}$$

where $Q_{ij}\equiv \langle \;\langle h_{i}h_{j}\rangle \;\rangle$ measures the correlations of external inputs with 〈〈·〉〉 denoting the average in a long time period for varying external inputs. This is the correlation-based learning model for feature map formation [27]. A feature map formation model should have a competition mechanism to prevent output neurons from having the same features as neighbors. The correlation-based model achieves the competition mechanism through negative components in *J* or *K* originating from inhibitory lateral connections.

**Figure 3:**
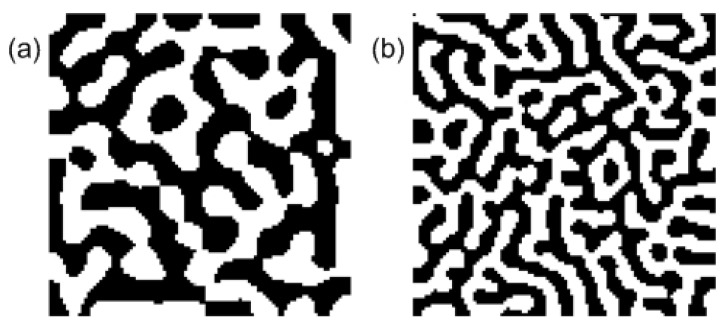
Ocular dominance map developed by the correlation-based learning model in Equation (31), where $K_{ij}=\left(1-kr_{ij}^{2}/\sigma^{2}\right)\exp\left(-r_{ij}^{2}/2\sigma^{2}\right)$ and $Q_{ij}=\delta_{ij}+\eta \left(1-\delta_{ij}\right)$ with σ=2, η=0.3, and (**a**) k=0.3 and (**b**) k=0.5. The size of input and output layers are given by 2 and 100×100, respectively. The difference between the feedforward connections from the two input neurons, corresponding to the left and the right retina ganglion cells, becomes the ocular dominance of output neurons.

Figure 3 shows the emergent feature map, developed by the correlation-based model. It is noteworthy that the feature map displays the same characteristics as the feature map observed in the primary visual cortex (V1) area. In simulations, the recursive lateral interaction *K* has been modeled as a Mexican-hat-shaped function of distance. The output neurons would have the same features (i.e., ocular dominance) as others if the degree of the competition, controlled by *k*, is sufficiently small [28]. In general, the visual input correlation matrix is diagonalized owing to the symmetry properties of external inputs; the feedforward connections is then represented by low-dimensional feature vectors or spin-like variables. For example, the ocular dominance of visual cortex neurons, based on the difference between feedforward connections from left and right eyes, can be represented by Ising-type spin variables. Consequently, the feature map formation in a visual cortex area can be explained in terms of the energy of a spin-like model [28,29,30,31,32].

The firing activity and the learning rule in the correlation-based model can be derived through the use of statistical mechanics. Suppose that the activity of output neuron *i* is determined probabilistically, so that its expectation value is expressed as

(32) $$\begin{array}{c} \langle u_{i}\rangle =\frac{1}{Z}\int Du\;u_{i}e^{-E[u]/T} \end{array}$$

with the partition function $Z\equiv \int Du\;e^{-E[u]/T}$ and a nonnegative constant *T*. Provided that *J* is a symmetric matrix, the energy function *E* is given by

(33) $$\begin{array}{c} E\left[u\right]=\frac{1}{2}\sum_{i,j\in G_{o}}D_{ij}u_{i}u_{j}-\sum_{i\in G_{o}}B_{i}u_{i} \end{array}$$

with $B_{i}\equiv \sum_{k\in G_{i}}W_{ik}h_{k}$. The expectation value is obtained from the derivative of the free energy F=−TlogZ with respect to the external source:

(34) $$\begin{array}{c} \langle u_{i}\rangle =\frac{T}{Z}\frac{\partial Z}{\partial B_{i}}=-\frac{\partial F}{\partial B_{i}}. \end{array}$$

Performing the Gaussian integral, we obtain the free energy as F=U−TS with the internal energy

(35) $$\begin{array}{c} U=-\frac{1}{2}\sum_{i,j}K_{ij}B_{i}B_{j}=-\frac{1}{2}\sum_{i,j,k,l}K_{ij}W_{ik}W_{jl}h_{k}h_{l} \end{array}$$

and the entropy

(36) $$\begin{array}{c} S=\frac{1}{2}\mathrm{tr}\log\left(G\right), \end{array}$$

where the connected two-point function $G_{ij}\equiv \langle u_{i}u_{j}\rangle -\langle u_{i}\rangle \langle u_{j}\rangle$ obtains the form G=TK. In accord, the expectation value in Equation (34) reduces to

(37) $$\begin{array}{c} \langle u_{i}\rangle =\sum_{k}K_{ik}B_{k}=\sum_{k,l}K_{ik}W_{kl}h_{l}, \end{array}$$

which agrees with Equation (29). Further, the learning rule in Equation (30) is obtained from the derivative of the free energy with respect to the connection strength:

(38) $$\begin{array}{c} \Delta W_{ij}\propto -\frac{\partial F}{\partial W_{ij}}=\sum_{k,l}K_{ik}W_{kl}h_{l}h_{j}. \end{array}$$

Equations (34) and (38) demonstrate that both the firing process and the learning process in a neural network can be derived from the free energy of the system. The derivative of the free energy with respect to the external source yields the neural activity in an extremum state. In comparison with the derivative of the energy function, the derivative of the free energy includes the effects of recursive interactions between neurons. Then, the derivative of the free energy with respect to the connection strength offers a relevant learning rule of the system. Nevertheless, the derivative of the entropy in Equation (36) exerts effects neither on the firing process nor on the learning rule. It is plausible that the entropy, related to autonomous neural firings via thermal fluctuations, exerts no effects on the neural firing process, although the informatix rule allows that the entropy maximization induces a proper competition mechanism in a neural network without inhibitory connections.

On the other hand, the pseudo-stochastic learning (PSL) model suggests that the entropy maximization principle would exert meaningful effects on the learning rule when the entropy is obtained not from the Boltzmann distribution but from the neural firing correlations in a Langevin equation [33]. We introduce a noise term to Equation (28) and write

(39) $$\begin{array}{c} \tau \frac{du_{i}}{dt}=-u_{i}+\sum_{j}J_{ij}u_{j}+\sum_{j}W_{ij}u_{j}+h_{i}+\xi_{i}, \end{array}$$

where $\xi_{i}$, referring to as endogenous neural firings via thermal fluctuations or external noisy currents, has the properties $\langle \xi_{i}\rangle =0$, $\langle h_{i}\xi_{j}\rangle =0$, and $\langle \xi_{i}\xi_{j}\rangle =\beta \delta_{ij}$ with constant β being proportional to the temperature *T*. The activities of input and output neurons in the steady state are given by $u_{i}=h_{i}+\xi_{i}$ and $u_{i}=\sum_{j}K_{ij}\left[\sum_{k}W_{jk}\left(h_{k}+\xi_{k}\right)+\xi_{j}\right]$, respectively, the expectation value of which agrees with Equation (29). The endogenous neural firings via thermal fluctuations exert no effects on the average over individual neural activities.

Meanwhile, the connected two-point function between output neurons becomes

(40) $$\begin{array}{c} G_{ij}=\beta {\left[KK^{†}+KWW^{†}K^{†}\right]}_{ij}, \end{array}$$

where $\beta KK^{†}$ (or $\beta KWW^{†}K^{†}$) corresponds to the neural correlations originating from the autonomous firings of output (or input) neurons. With the substitution $G\approx TKWW^{†}K^{†}$ in Equation (36), the gradient flow of the free energy in Equation (38) leads to the learning rule

(41) $$\begin{array}{c} \Delta W_{ij}\propto {\left[KWQ+T{\left(WW^{†}\right)}^{-1}W\right]}_{ij}, \end{array}$$

where ${\left(WW^{†}\right)}_{ij}$ is the inner product between incoming connection vectors onto output neurons *i* and *j*, so that ${\left(WW^{†}\right)}^{-1}W={\left(I-I+WW^{†}\right)}^{-1}W\approx \left(I-WW^{†}\right)W$ hinders output neurons from having the same feedforward connection pattern as others. Note that the second term in this equation corresponds to the first term in Equation (27) because $\frac{1}{2}\mathrm{tr}\log\left(WW^{†}\right)=\mathrm{tr}\log\left(W\right)$ for a square matrix *W*.

**Figure 4:**
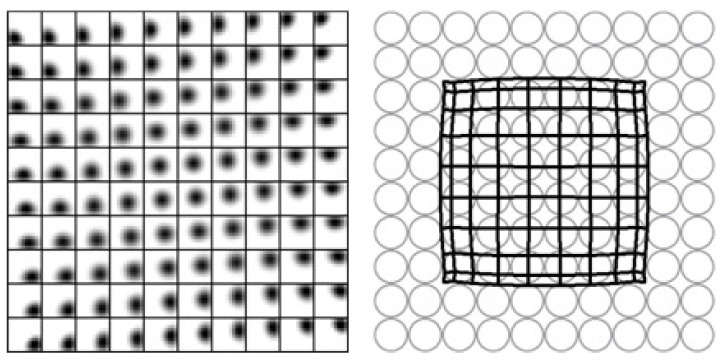
Topographic map developed by the PSL model in Equation (41). The **left** figure shows the receptive field (RF) of output neurons on the input layer, where the small boxes and the axes represent the connection strengths from input neurons to individual output neurons and the neuronal positions in the output layer, respectively. The **right** figure depicts the topographic map obtained from the connection strengths, where the gray circles and the mesh points represent, respectively, the positions of the input neurons and the RF centers of the output neurons on the input neurons. We have taken $K_{ij}=\exp\left(-r_{ij}^{2}\right)$, $Q_{ab}=\exp\left(-r_{ij}^{2}\right)$, and T=0.1, along with the approximation ${\left(WW^{†}\right)}^{-1}\approx I-WW^{†}$ and the normalization condition ${\left(WW^{†}\right)}_{ii}=1$. Both the input and the output layers have the size 10×10.

Figure 4 presents a feature map, developed by the PSL model [33]. It has characteristics of a topographic map, such as the well-ordered connection distribution from retina (or LGN) cells to V1 neurons. In simulations, the lateral connections *J* (or *K*) have no negative values, but the entropy-originating term brings about adequate competition between output neurons.

## 4. Feynman Machine

The Feynman machine is a neural network model based on the free energy principle [34,35,36,37]. Both the firing and the learning rule in the model are derived from a single free energy, which is defined via the Feynman path integral rather than from the Boltzmann distribution. The typical form of a Feynman machine bases on an approximate solution of the firing dynamics equation in a biological neuron model:

(42) $$\begin{array}{c} C\frac{dV_{i}}{dt}=I_{i}^{\mathrm{syn}}+I_{i}^{\mathrm{ion}}+I_{i}^{\mathrm{ext}}, \end{array}$$

where $V_{i}\left(t\right)$ is the membrane potential of neuron *i* at time *t* and *C* the capacitance per unit surface area. On the right-hand side, $I_{i}^{\mathrm{syn}}$, $I_{i}^{\mathrm{ion}}$, and $I_{i}^{\mathrm{ext}}$ represent the synaptic, ionic, and external stimulus currents, respectively. The synaptic current is given by

(43) $$\begin{array}{c} I_{i}^{\mathrm{syn}}\left(t\right)=\sum_{j}\int dt^{\prime }g_{ij}^{\mathrm{syn}}\left[V_{ij}^{\mathrm{syn}}-V_{i}\left(t\right)\right]\;\alpha \left(t-t^{\prime }\right)\;\phi_{j}\left(t^{\prime }\right), \end{array}$$

where $g_{ij}^{\mathrm{syn}}$ is the maximum conductance per unit surface area (with $g_{ii}^{\mathrm{syn}}\equiv 0$), $V_{ij}^{\mathrm{syn}}\equiv V_{ij}^{\mathrm{syn}}-V^{\mathrm{eq}}$ is the synaptic reversal potential $V_{ij}^{\mathrm{syn}}$ of the synaptic connection from neuron *j* to neuron *i* measured from the rest membrane potential $V^{\mathrm{eq}}$, and $V_{i}\left(t\right)=V_{i}\left(t\right)-V^{\mathrm{eq}}$ is the membrane potential of neuron *i* from the rest potential. The function α(t) is typically modeled as α(t)=g(t)exp[−g(t)] with $g\left(t\right)\equiv \max[\left(\tau_{d}-t\right)/\tau_{c},0]$, where $\tau_{d}$ is the delay time and $\tau_{c}$ the characteristic time. The ionic current is taken to be of a variety of form on the model-by-model basis.

It is rather formidable to obtain the exact solution of the firing dynamics equation in most models; however, in consideration of that $V_{i}\left(t\right)$ would converge to $V^{\mathrm{eq}}$ in the absence of external inputs and synaptic connections, we express the solution as a series in ϕ:

(44) $$\begin{array}{c} V_{i}\left(t\right)=h_{i}\left(t\right)-\sum_{j}\int dt^{\prime }D_{ij}^{+}\left(t-t^{\prime }\right)\phi_{j}\left(t^{\prime }\right)+O\left(\phi^{2}\right). \end{array}$$

Here, $h_{i}\left(t\right)$ relates to the leaky integration of external stimulus current and $D_{ij}^{+}\left(t\right)\approx -\lambda^{\mathrm{syn}}\left(t\right)W_{ij}$ represents the synaptic interactions with other neurons, where

(45) $$\begin{array}{c} W_{ij}\equiv g_{ij}^{\mathrm{syn}}V_{ij}^{\mathrm{syn}}=g_{ij}^{\mathrm{syn}}\left(V_{ij}^{\mathrm{syn}}-V^{\mathrm{eq}}\right) \end{array}$$

is the synaptic connection strength from neuron *j* to neuron *i* and the overall strength $\lambda^{\mathrm{syn}}\left(t\right)=0$ for t<0. The exact form of $h_{i}\left(t\right)$ and $\lambda^{\mathrm{syn}}\left(t\right)$ is obtained theoretically for simple biological neuron models such as the leaky integrating -firing model or via simulations [36]. The synaptic connection strength $W_{ij}$ becomes excitatory for $V_{ij}^{\mathrm{syn}}\geq V^{\mathrm{eq}}$ and inhibitory otherwise. It is usually not the reversal potential $V_{ij}^{\mathrm{syn}}$ but the maximum conductance $g_{ij}^{\mathrm{syn}}$ (≥0) which is biologically plastic. Accordingly, the sign of a synaptic connection strength does not alter in the learning process.

While the variable $\phi_{i}\left(t\right)$ can take different values in different trials owing to stochasticity, the expectation value $\langle \phi_{i}\left(t\right)\rangle$ tends to increase with the membrane potential $V_{i}\left(t\right)$. Considering that a neuron cannot fire again during the refractory period $\tau_{r}$, we take the firing probability to be $\langle \phi_{i}\left(t\right)\rangle \propto \exp\left[T^{-1}\left\{V_{i}\left(t\right)-V^{\mathrm{th}}+\int dt^{\prime }\lambda^{\mathrm{rep}}\left(t-t^{\prime }\right)\phi_{i}\left(t^{\prime }\right)\right\}\right]$ or

(46) $$\begin{array}{c} \langle \phi_{i}\left(t\right)\rangle \propto \exp\left[\frac{1}{T}\left\{-\sum_{j}\;\int \;dt^{\prime }\;D_{ij}^{+}\left(t-t^{\prime }\right)\phi_{j}\left(t^{\prime }\right)+B_{i}\left(t\right)\right\}\right], \end{array}$$

where $D_{ij}^{+}\left(t\right)=-\delta_{ij}\lambda^{\mathrm{rep}}\left(t\right)+D_{ij}^{+}\left(t\right)$ and $B_{i}\left(t\right)=h_{i}\left(t\right)-V^{\mathrm{th}}$ with $V^{\mathrm{th}}\equiv V^{\mathrm{th}}-V^{\mathrm{eq}}$ being the threshold membrane potential from the rest potential. Here, $-\lambda^{\mathrm{rep}}\left(t\right)$ takes into account the presence of the refractory period, either diverging (→∞) for $0<t\leq \tau_{r}$ or vanishing (→0) otherwise. Note that $D_{ij}^{+}\left(t\right)$, being applicable only for t>0, represents the neural interactions along the ordinary time direction. The constant *T*, playing the role of temperature, measures the intensity of the noisy current [38,39].

**Figure 5:**
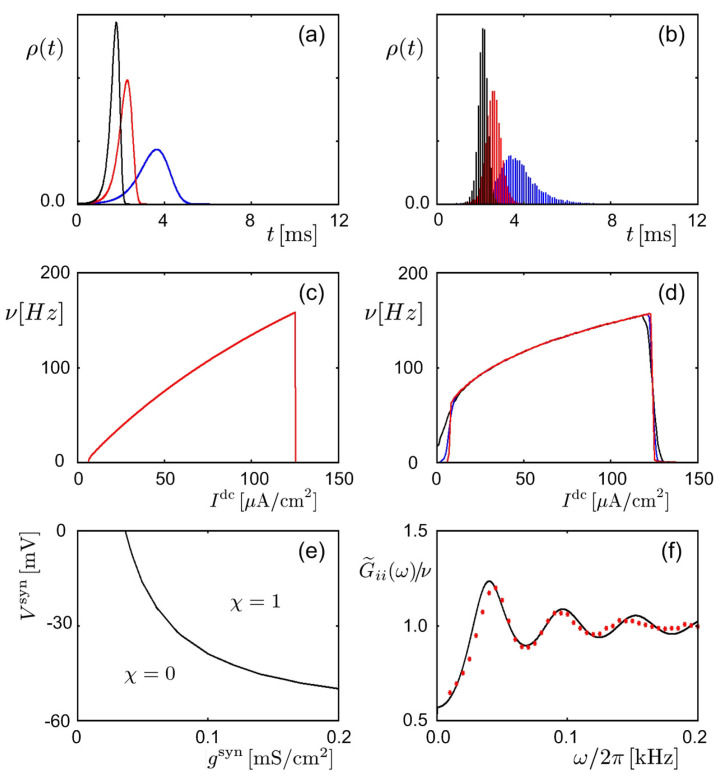
Neural firing statistics in the Hodgkin–Huxley model, predicted via the Feynman machine and obtained via simulations. (**a**,**b**) Firing probability ρ(t) (≡〈ϕ(t)〉) of a neuron versus time *t*, activated by a presynaptic neuron fire at t=0 in the presence of external noisy currents. Results from (**a**) theory and (**b**) simulations for three different values of the connection strength are presented in black, red, and blue [35]. (**c**,**d**) Mean firing rate $\nu \equiv \Delta^{-1}\int_{0}^{\Delta }dt\;\rho \left(t\right)$, with a long time interval Δ, of a neuron versus external direct current $I^{\mathrm{dc}}$ given additionally to activate the neuron. Shown are results from (**c**) theory and (**d**) simulations, for three different values of the noisy current intensity plotted in black, red, and blue lines in (**b**) [36]. (**e**) Plot of the contour line χ=0.5, obtained via simulations, on the parameter plane $\left(g^{\mathrm{syn}},V^{\mathrm{syn}}\right)$, where $\chi \equiv \int_{0}^{\tau_{r}}dt\;\rho \left(t\right)$, with $\tau_{r}$ being the refractory period, is the total firing probability of a neuron activated by the synaptic current from a presynaptic neuron via a synapse of $\left(g^{\mathrm{syn}},V^{\mathrm{syn}}\right)$. Theoretically, the contour line consists of the points on which $g^{\mathrm{syn}}\left(V^{\mathrm{syn}}-V^{\mathrm{eq}}\right)$ is constant [40]. (**f**) Frequency dependence of ${\tilde{G}}_{ii}\left(\omega \right)/\nu$, where ${\tilde{G}}_{ii}\left(\omega \right)$ is the Fourier transform of the autocorrelation function $G_{ii}\left(t\right)$. The black line represents theoretical results, whereas the red dots plot data points obtained via simulations [35].

Equation (46) makes it possible to calculate easily the firing probability of a neuron in a short time interval for given conditions. As an example, suppose that neuron *i* receives synaptic currents from other neurons in the absence of external stimulus currents. In the limit T→0, the firing condition of the neuron may be expressed as

(47) $$\begin{array}{c} \max\left[\sum_{j}\lambda^{\mathrm{syn}}\left(t-t_{j}^{*}\right)W_{ij}\right]\geq V^{\mathrm{th}}, \end{array}$$

where $t_{j}^{*}$ represents the recent firing timing of neuron *j* [40]. The contour line in Figure 5e, obtained from $W_{ij}=g_{ij}^{\mathrm{syn}}\left(V_{ij}^{\mathrm{syn}}-V^{\mathrm{th}}\right)$ being constant in Equation (47), thus illustrates the condition of synaptic connection for a postsynaptic neuron to fire in response to the firing of a single presynaptic neuron. Note that biological neurons may not vary the activity in a short time because neural firing spikes have nearly the same form. Instead, they can perform a function or a communication through the use of the sensitivity for spike timings on the time scale of milliseconds [14,15]. Equation (47) describes how a biological neuron can have the sensitivity for exact timings of input spikes.

However, Equation (46) or Equation (47) does not allow one to predict the firing probability on a long-time scale during which neurons may interact recursively with one another. Such statistics of neural firings in a long time can only be obtained after the effects of all possible neural interaction ways are taken into account. The Feynman machine solves this problem by describing neural interactions in the form of a Feynman path integral. Specifically, the one-point function of neural firing states is given by

(48) $$\begin{array}{c} \langle \phi_{i}\left(t\right)\rangle =\frac{1}{Z}\mathrm{Tr}\;\phi_{i}\left(t\right)\;e^{A[\phi ]/T} \end{array}$$

with the partition function $Z\equiv \mathrm{Tr}\;e^{A[\phi ]/T}$ as the normalization factor. Here, the trace stands for the summation over all configurations of firing states at the grid points in the spatiotemporal space. The action is given by

(49) $$\begin{array}{c} A\left[\phi \right]=-\frac{1}{2}\sum_{i,j}\int dtdt^{\prime }D_{ij}\left(t-t^{\prime }\right)\phi_{i}\left(t\right)\phi_{j}\left(t^{\prime }\right)+\sum_{i}\int dtB_{i}\left(t\right)\phi_{i}\left(t\right) \end{array}$$

with $D_{ij}\left(t\right)\equiv D_{ij}^{+}\left(t\right)+D_{ji}^{+}\left(t\right)$. Precisely speaking, the one-point function in Equation (48) contains the effects of interactions with firings at times later than the observation time, so that the firing probability in Equation (46) can be obtained after non-physical interactions are eliminated or the second quantization technique is adopted. The one-point function can be rewritten in the form

(50) $$\begin{array}{c} \langle \phi_{i}\left(t\right)\rangle =-\frac{\partial F}{\partial B_{i}\left(t\right)} \end{array}$$

with the free energy F=−TlogZ. The connected two-point function $G_{ij}\left(t,t^{\prime }\right)\equiv {\langle \phi_{i}\left(t\right)\phi_{j}\left(t^{\prime }\right)\rangle }_{c}\equiv \langle \phi_{i}\left(t\right)\phi_{j}\left(t^{\prime }\right)\rangle -\langle \phi_{i}\left(t\right)\rangle \langle \phi_{j}\left(t^{\prime }\right)\rangle$ also obtains the form

(51) $$\begin{array}{c} G_{ij}\left(t,t^{\prime }\right)=-\frac{T\partial^{2}F}{\partial B_{i}\left(t\right)\partial B_{j}\left(t^{\prime }\right)}. \end{array}$$

The Feynman machine makes it possible to predict the firing probability or the cross-correlation on the millisecond time scale, with help of the methods in, e.g., liquid theory and statistical or quantum field theory [35]. For instance, after non-physical interactions are eliminated, the firing probability of neuron *i* at time *t* is expressed in the form

(52) $$\begin{array}{c} \langle \phi_{i}\left(t\right)\rangle =z_{i}\left(t\right)+\sum_{j}\int dt^{\prime }z_{i}\left(t\right)z_{j}\left(t^{\prime }\right)f_{ij}^{+}\left(t-t^{\prime }\right)+\frac{1}{2}\sum_{j,k}\int dt^{\prime }dt^{\prime \prime }z_{i}\left(t\right)z_{j}\left(t^{\prime }\right)z_{k}\left(t^{\prime \prime }\right)\\ \;\times \left[2f_{ij}^{+}\left(t-t^{\prime }\right)f_{jk}\left(t^{\prime }-t^{\prime \prime }\right)+f_{ij}^{+}\left(t-t^{\prime }\right)f_{ik}^{+}\left(t-t^{\prime \prime }\right)+f_{ij}^{+}\left(t-t^{\prime }\right)f_{ik}^{+}\left(t-t^{\prime \prime }\right)f_{jk}\left(t^{\prime }-t^{\prime \prime }\right)\right]+\ldots , \end{array}$$

where $z_{i}\left(t\right)\equiv e^{B_{i}\left(t\right)/T}$ and $f_{ij}\left(t\right)\equiv f_{ij}^{+}\left(t\right)+f_{ji}^{+}\left(-t\right)$ with $f_{ij}^{+}\left(t\right)=e^{-D_{ij}^{+}\left(t\right)/T}-1$ correspond, respectively, to the fugacity and the Mayer function in statistical physics. Some typical results predicted via the Feynman machine are presented in Figure 5, which displays results from simulations as well.

## 5. Learning Principle in the Feynman Machine

Although the Feynman machine can adopt any kind of synaptic plasticity rule in its learning process, the minimization of the path-integral free energy serves as an ideal learning rule in the Feynman machine. Namely, the desirable change in a synaptic coupling strength is governed by the gradient flow of the free energy:

(53) $$\begin{array}{c} \Delta W_{ij}\propto -\frac{\partial F}{\partial W_{ij}}. \end{array}$$

The Feynman machine learning principle is convinced by the fact that the firing states and the connection states constitute the coupled dynamics of a neural system with different attributes. The partition function $Z=\mathrm{Tr}e^{A[\phi ]/T}$ can be rewritten in the form

(54) $$\begin{array}{c} Z=\prod_{i,t}\int_{-\infty }^{\infty }d\phi_{i}\left(t\right)\delta \left(\phi_{i}\left(t\right)\left(\phi_{i}\left(t\right)-1\right)\right)e^{A[\phi ]/T}\equiv \int D\phi \;W_{a}\left[\phi \right]\;e^{A[\phi ]/T}. \end{array}$$

Accordingly, in consideration of varying external inputs, the partition function of a Feynman machine on a long time scale takes the form

(55) $$\begin{array}{c} Z_{L}=\int D\phi \;DW\;Dh\;W_{a}\left[\phi \right]\;W_{s}\left[W\right]\;W_{e}\left[h\right]\;e^{A[\phi ,W]/T}, \end{array}$$

where $W_{a}$, $W_{s}$, and $W_{e}$ are the weights or probability functions for neural activity, synaptic coupling strength, and external inputs, respectively. The connection state at the end of the learning process then obtains the form

(56) $$\begin{array}{c} \langle W_{ij}\rangle =\;\frac{1}{Z_{L}}\;\int \;D\phi DWDhW_{a}\left[\phi \right]W_{s}\left[W\right]W_{e}\left[h\right]W_{ij}e^{A[\phi ,W]/T}. \end{array}$$

With $F_{L}\equiv -\log Z_{L}$, the equilibrium condition $\partial F_{L}/\partial W_{ij}=0$ brings on

(57) $$\begin{array}{c} \int DhW_{e}\left[h\right]\frac{\partial Z}{\partial W_{ij}}=0, \end{array}$$

which can be achieved by the gradient flow in Equation (53).

The Feynman machine needs an additional rule to restrict the synaptic coupling strengths within a range because the minimization of the free energy is achieved just by increasing the excitatory connection strengths and/or decreasing the inhibitory connection strengths as usual. One can define the weight function $W_{s}\left[W\right]$ to control the synaptic coupling strength; however, the general form of $W_{s}$, which works properly in extensive circumstances and bases on biological experiments, is yet to be suggested.

Remarkably, the typical form of the biological synaptic plasticity emerges from this ideal learning rule in the Feynman machine. With the STDP rule in Equation (5) substituted into Equation (53), the STDP window obtains the form

(58) $$\begin{array}{c} \Omega_{ij}\left(t\right)\propto -\frac{\partial^{2}A}{\partial C_{ij}\left(t\right)\partial W_{ij}}. \end{array}$$

**Figure 6:**
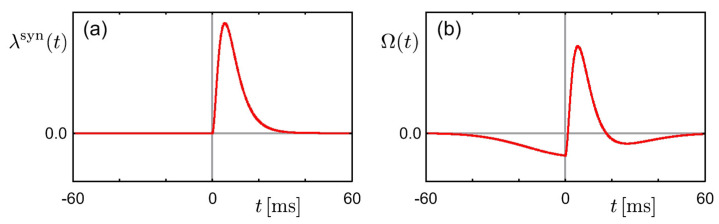
Plots of (**a**) $\lambda^{\mathrm{syn}}\left(t\right)$ and (**b**) Ω(t), where $\lambda^{\mathrm{syn}}\left(t\right)$ is taken from the solution of a leaky integrating–firing model, given by $C^{-1}\int_{0}^{t}dt^{\prime }e^{\gamma (t^{\prime }-t)}\alpha \left(t^{\prime }\right)$, and $\Sigma (t,t^{\prime })$ is modeled simply as $A\exp[-{\left(t+t^{\prime }\right)}^{2}/2\sigma^{2}-{\left(t-t^{\prime }\right)}^{2}/2\sigma^{2}]$ with A=0.02 and σ=30 ms.

A lengthy but straightforward calculation leads the STDP window to take the form

(59) $$\begin{array}{c} \Omega_{ij}\left(t\right)\propto \lambda^{\mathrm{syn}}\left(t\right)-\int dt^{\prime }\Sigma_{ij}\left(t,t^{\prime }\right)\lambda^{\mathrm{syn}}\left(t^{\prime }\right), \end{array}$$

where $\Sigma_{ij}\left(t,t^{\prime }\right)\equiv T\partial^{2}S/\partial C_{ij}\left(t\right)\partial D_{ij}\left(t^{\prime }\right)$ with the entropy $S\approx \frac{1}{2}\mathrm{tr}\log G$. The first term on the right-hand side of Equation (59), originating from the derivative of the internal energy $U=\left(1/2\right)\sum_{i,j}\int dtD_{ij}\left(t\right)C_{ij}\left(t\right)$, produces LTP for pre-before-post pairings. The second term, originating from the derivative of the entropy, produces LTD for post-before-pre parings and occasionally LTP for post-before-pre pairings. The resulting time dependence of $\lambda^{\mathrm{syn}}\left(t\right)$ and Ω(t) is shown in Figure 6. It is pleasing that these results indeed coincide with the form of the STDP window as well as the LTP part, observed in experiment.

Thus far, the learning process in a biological neural system has been predicted through estimating the cross-correlations of neural firings on given circumstances and producing it with a STDP window which is modeled rather coarsely from experimental observations [32,41,42]. On the other hand, the Feynman machine learning rule makes it possible to derive the cross-correlations, the STDP window, and their products from the derivatives of a free energy. For instance, the Feynman machine learning rule applied to the input–output layer system yields the development of a feedforward connection in the form [37]

(60) $$\begin{array}{c} \Delta W_{ij}\propto \int d\omega {\left[\tilde{K}\left(\omega \right)W\tilde{Q}\left(\omega \right)\right]}_{ij}-\beta {\left[{\left(WW^{†}\right)}^{-1}W\right]}_{ij}, \end{array}$$

where ${\tilde{K}}_{ij}\left(\omega \right)$ and ${\tilde{Q}}_{ij}\left(\omega \right)$ are the Fourier transforms of the vertex two-point function $K_{ij}\left(t\right)$ between output neurons and the input correlation function $Q_{ij}\left(t-t^{\prime }\right)\equiv T^{-2}\int Dh\;W_{e}\left[h\right]h_{i}\left(t\right)h_{j}\left(t^{\prime }\right)=T^{-2}\langle \;\langle h_{i}\left(t\right)h_{j}\left(t^{\prime }\right)\rangle \;\rangle$ in the spatiotemporal space.

This learning rule is capable of recognizing temporal patterns, such as a STDP-based learning model. It is not a firing-rate-based learning model but a spiking-timing-based one that can explain the development of selectivity for moving visual images, such as the directional selectivity in V1 [32]. If external inputs have weak correlations on the fast time scale so that $\tilde{Q}\left(\omega \right)$ has a large peak at ω=0, Equation (60) reduces to a firing-rate-based learning model expressed in the form

(61) $$\begin{array}{c} \Delta W_{ij}\propto {\left[\tilde{K}\left(0\right)W\tilde{Q}\left(0\right)\right]}_{ij}-\beta {\left[{\left(WW^{†}\right)}^{-1}W\right]}_{ij}, \end{array}$$

which corresponds just to Equation (41) in the PSL model.

## 6. Discussion

It is crucial in theoretical neuroscience to develop an abstract neuron model without losing the essential features of the biological neurons for information processing or learning. It is informative that Boolean algebra, rather than the real behavior of electronic devices, plays a key role in understanding how a computer operates. In a similar sense, the model neurons could be more significant than real neurons in understanding how the brain works. A neuron model explaining the firing activity and the learning rule through the concepts and principles in statistical mechanics could be helpful for revealing the essence of neural computing and learning mechanism.

The modeling based on statistical mechanics gives several advantages over other modeling methods. The firing activity and the learning rule are described by differential equations, the outcomes of which are inferred from the extremum states of appropriate functions. For example, the Hopfield network shows how to store desired patterns as the extremum states in a neural system. The probabilistic firing rule, based on important sampling, is regarded as a suitable way to avoid trapping in a local minimum during the learning process.

Nevertheless, it is an intriguing problem to probe the neural system via (equilibrium) statistical mechanics. The firing process in a biological neural network is usually governed by (non-integrable) nonlinear dynamical equations, and the learning process is usually taken under non-stationary external stimuli. A standard way to express the neural process in terms of statistical mechanism is to define the energy of the system. In view of statistical mechanics, this approach is based on some assumptions about the neural system, such as the ergodic hypothesis, indifference principle, entropy maximization principle, and so on. In particular, the assumption of ergodicity allows that a neural system is described by an energy function with no explicit time variable. Such an energy function further satisfies the invariance condition under the exchange of element positions, so that interaction strength between two elements should be symmetric. Therefore, with the connection strength between neurons regarded as the interaction strength between them, the energy-based neuron model assumes all connections to be symmetric and treat asymmetric connections as part of external inputs. Stochastic equations used to describe various phenomena subject to thermal fluctuations offer a possible way to describe the process in a neural system with asymmetric connections; the Langevin equation description of the firing-rate neuron model provides an example. The adoption of an explicit time variable could be another way to handle the problem. Namely, in the Feynman path integral approach, neural processes are described on the basis of an action, which is expressed as an integral in the spatiotemporal space. The action could satisfy the invariance condition if the interaction strength between neural pairs are given by a symmetric function in the spatiotemporal space or a Hermitian function in the momentum space.

An important aspect in a neural network model based on statistical mechanics is that the entropy maximization principle may be the origin of the competitive learning mechanism, which is indispensable for the learning process to prevent neurons from having the same features or functions with neighbors. In most neuron models, the implementation of the competition mechanism is based on the existence of inhibitory connections [27] or a normalization condition for neural activity [43], among others. Although not all statistical-mechanics-based models implement the competition mechanism through the maximization, the gradient descent in entropy does induce proper competitive learning behavior in a neural network. Particularly in a biological neural network, the competitive learning mechanism turns out to be based on the entropy maximization principle.

The statistics of neural firings could be obtained from the derivatives of the free energy with respect to external inputs if the free energy of a neural system is well defined. The learning rule in a statistical-mechanics-based model is often defined as the gradient descent in several quantities. However, the free energy is the most natural quantity to define an ideal learning process. The process in a neural system is basically given by the change in firing and connection states occurring on different dynamics scales. Namely, the neural process is described by dynamics coupled in variables with two different attributes, so that the extremum states of the free energy should relate to the outcomes of not only the firing but also the learning process.

A statistical-mechanics-based model also opens the possibility of understanding various phenomena in a neural system in terms of the general theory of phase transitions. The change in the features of rhythmic firings may be interpreted as a kind of phase transition phenomena. Further, the frame of connection structure could be altered in the learning process, depending on relevant conditions and temperature. In many learning models, the architecture is regarded as a fixed one and the change of connections is allowed without modifying the basic frame. Such models often suffer from the failure in the learning process when the architecture is not proper to handle given learning data. On the other hand, in a biological neural system, the architecture should not be fixed but modifiable flexibly; otherwise, a neural system, such as the brain, cannot operate steadily under various environmental conditions. It has been shown that a STDP-based learning model can modify the number and sizes of layers in a multilayer system, where the competition between connections causes extinction of less important connections and reorganization of the connection structure [8,44].

The Feynman machine allows one to interpret many aspects of the neural firing and learning processes in terms of general concepts and principles in statistical mechanics. While the Feynman machine has descended from conventional statistical-mechanics-based neuron models, it adheres to several attributes of neural processes. Among those, the explicit time variable, which has been neglected in conventional neural network models, plays a key role since the computing or learning mechanism in a biological neural network is often performed depending on exact firing timings of neurons. Unlike the original version which ignores such attributes of real neural processes as the effects of the variance in the membrane potential during neural firing, the Feynman machine describes neural dynamics in terms of the firing timing instead of the membrane potential, so that it can represent more realistic neural dynamics, as the relation between the membrane potential and firing timing in Equation (44) becomes more completely characterized. In particular, it has been demonstrated that an extended version of the Feynman machine with an additional interaction term is needed for figuring out neural firing dynamics in a synchronized state because the exact form of the membrane potential during neural firing may exerts effects on the phenomena [36].

Finally, we remark that the Feynman machine can be applied to explaining the emergence of computing ability not only in a neural system but also in a physical system. If the dynamics of the physical system is expressed in the form of a path integral involving several types of dynamical variables with the rate of change on different time scales and the changes of the variables optimize the path integral, the system can perhaps acquire the ability of computing, memorizing, or learning.
